# Exploring dose-dependent outcomes of photobiomodulation with two infrared wavelengths applied to injured *Drosophila* retinae

**DOI:** 10.1371/journal.pone.0354797

**Published:** 2026-08-19

**Authors:** Michael Meece, Stephen Worthington, Lawrence Pierce, Maryia Sikirzhytskaya, Elke K. Buschbeck

**Affiliations:** 1 Department of Biological Sciences, University of Cincinnati, Cincinnati, Ohio, United states of America; 2 Butler University, Indianapolis, Indiana, United states of America; Dartmouth Hitchcock Medical Center, UNITED STATES OF AMERICA

## Abstract

The use of photobiomodulation as a therapeutic tool has attracted copious research over the past several years. However, given the many variables involved in the design of a treatment regimen, such as wavelength, irradiance, exposure duration, number of treatments, and total treatment period, many studies are inherently not comparable as they often differ in at least one of these parameters. Here, we assess dose and wavelength dependency of photobiomodulation in a model of light-induced retinal damage in *Drosophila melanogaster*. Light-damaged *Drosophila* were exposed to either 850 or 950 nm light twice per day over a 10-day period with a total dose of 1, 4, 11, or 20 J/cm^2^. Treatment with 850 nm light was found to be generally more effective in promoting recovery of photoreceptor function, with higher doses being more efficacious. Treatment with 950 nm light, regardless of dose, did not result in improved photoreceptor function; however, a slightly increased photoreceptor response was observed upon treatment with a fluence of 11 J/cm^2^ when compared to other doses of the same wavelength. We also observed elevated ATP levels in 850 nm treated flies and higher doses of 950 nm light. Our findings suggest that increased ATP did not directly correlate with photoreceptor recovery, implying that multiple processes are involved, the nature of which warrant further investigation.

## Introduction

In recent years, near-infrared (NIR) light has gained growing attention as a potential therapeutic approach for disorders and injuries of the nervous system [1–3]. Most interestingly, there have been studies that showed amelioration of damage and stress in cells and tissues by affecting metabolic activity, with subsequent improved recovery through the application of NIR [4, 5]. These findings have considerable therapeutic potential; however, studies tend to use very different paradigms and often quantify light in ways that are non-convertible, preventing the ability of inter-study comparisons. This obfuscates which parameters work best within this very complex parameter space of ways to apply IR light. Based on previous studies [4, 6, 7], including one of our own group [8], IR light of the wavelengths 850 and 950 nm are particularly interesting because these wavelengths may affect damaged tissues differently, but it is unclear to what extent differences in intensity may have influenced previous findings. To address this gap in knowledge, we aim to provide a direct systematic comparison of different doses of NIR light of these two key wavelengths. Drosophila compound eyes are a well-established model system for investigating tissue damage and recovery [9]. Their eyes are made up of glia-like support cells and neuronal photoreceptor cells [10]. The latter has a stereotyped, measurable response to light exposure, which we have leveraged to establish light-induced retinal damage and subsequent wavelength-specific IR-assisted regeneration [8]. Photoreceptors are also energetically costly, relying heavily on mitochondria, which in themselves are characterized by deeply conserved pathways [11].

Although increasing evidence highlights the potential for NIR photobiomodulation (PBM) as a useful tool in therapeutic treatment, especially of neural degeneration and reduction of stress within a tissue [3], much debate remains on how such treatments work. Many studies point towards improved mitochondrial function [4, 6, 12], with one proposed mechanism suggesting that cytochrome C oxidase (COX), a key enzyme of the electron transport chain of the mitochondria, is ultimately responsible for the effect of many PBM paradigms. Specifically, it is thought that the light-absorbing metal centers of COX are photosensitive [13]. Absorbed light increases mitochondrial membrane potential, thus allowing for greater buildup of the proton gradient in the mitochondrial intermembrane space [14, 15]. This then results in a greater yield of ATP [12]. Additional proposed mechanisms exist, for example: it has been postulated that longer wavelengths of light, specifically those closer to >1000 nm, where light is more readily absorbed by molecular water, might result in increased temperature and ultimately a faster metabolic rate [16]. NIR light may also influence growth factors [17] and enhance cell proliferation [18].

Despite the discovery of so many exciting different possible mechanisms, the field of photobiomodulation has struggled to find synthesis and consensus. As already outlined, the field is hampered by incompatible experimental approaches that are due to a very complex related parameter space. This includes different ways to quantify light, differences in applied wavelengths and dosages, differences in the treatment paradigm such as including the application of continuous or pulsed light, as well as differences at what time in the day/night cycle lights are applied. In addition, even if the same peak wavelengths were applied, the type of light that was used still affects the exact specification of the light, as parameters such as half-width of the wavelength’s peak and coherence of the emitted light can also differ. Finally, of course, outcomes can relate to differences in the study system.

Confounding things further, researchers in different sub-disciplines often express their light measurements in different units. This is especially problematic as radiometric, quantum radiometric, and photometric units are not directly translatable [19]. For example, lux is inherently biased toward human visual sensitivity, whereas radiometric and quantum radiometric units are more biologically relevant, owing to the absorption of photons of specific wavelengths and energies, measured either as photon flux or as energy [19]. Notably, radiometric and quantum radiometric units are only interconvertible if a spectrum is available [19], which often is not the case. Furthermore, PBM often uses different wavelengths that, even if applied at comparable intensity, could elicit differing outcomes, possibly due to different underlying mechanisms. For example, we previously found that exposure to comparable photon fluxes of NIR light of different wavelengths following light-induced retinal damage resulted in varying outcomes, with exposure to 850 nm having the most beneficial outcome on photoreceptor function and ATP production [8]. Treatment with 950 nm light did not positively affect photoreceptor function, but it did increase ATP production and led to morphological improvements [8]. Additionally, another study found that exposure to 750 and 950 nm light both reduced COX activity and oxygen consumption, whereas 810 nm light did the opposite, resulting in increased COX activity [4]. Regardless of the wavelength or units used to measure light, many studies assess the efficacy of light treatment under vastly different intensities that vary from dim illumination to very bright lighting, as well as using either pulsed laser or LED exposure, and the duration of treatment and number of doses given during treatment also fall across a wide array [2]. This highlights how, even though the commonly used measurement of fluence is a very useful way to characterize light exposure, it is still insufficient if used exclusively. To address this, and in line with what should become standard practice in the field, we quantify our light stimuli using a variety of units.

Following our previous work [8], we use *D. melanogaster* as a preferred study organism. This well-established model organism for many human diseases [20] is particularly well suited to the field of PBM owing to its genetic tangibility and short lifecycle, and the fact that it offers a good readout for fundamental biological processes, as PBM likely is based on deeply conserved mechanisms [21]. In addition, the small size makes it easier to effectively apply infrared irradiation. It has been shown that insect cuticle is quite capable of transmitting infrared light, particularly at therapeutic wavelengths [22, 23]. Given the intense metabolic demand of the Dipteran eye and brain [24], and the specific effects of PBM on metabolic activity, *Drosophila* makes for a comprehensive study system in this regard. We have previously shown that NIR treatment was effective in ameliorating light-induced retinal degeneration in White mutant *Drosophila melanogaster*, with improvements in electrophysiological function, retinal structure, and ATP production [8]. This previous study compared light in terms of photon count, rather than total energy, leaving some ambiguity regarding dose- dependent effects. Here we hypothesize that it is the wavelength itself that leads to a difference in outcome, rather than the intensity. To resolve this question, we here investigate the effects of dosage, to allow for a more detailed comparison of 850 and 950 nm light. Among other intensities, we specifically contrast these wavelengths both, in terms of comparable photon counts and as identical power densities. The latter necessarily involves different photon fluxes, owing to the relationship between photon wavelength and energy [19]. With this comprehensive comparison we aim to gain a more comprehensive relationship between dose and treatment outcome and to gain insights on how to optimize PBM for these wavelengths.

## Methods

### *Drosophila* husbandry

**Fig 1:**
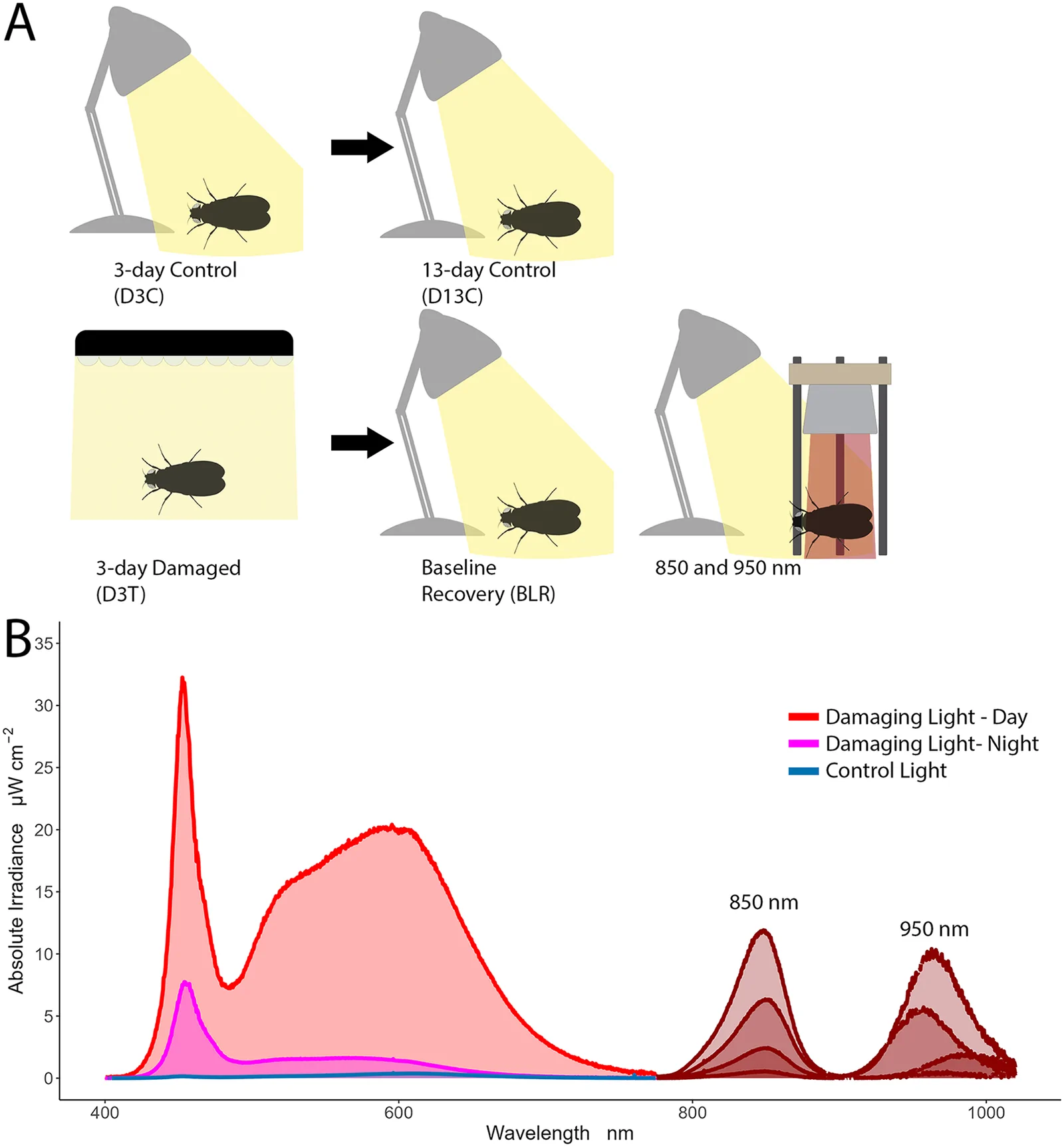
Light Exposure Paradigm. A) Diagram of experimental design of light treatment. D3T flies were exposed to constant bright light for 72 h to induce light damage. To assess recovery with infrared light, they were placed in follow-up treatments for 10 days. These consisted of controlled background lighting environments that were identical to those of undamaged control flies, either without (BLR) or with the addition of 850 or 950 nm IR light (NIR-treated). This image is modified from Fig 1A in Meece et al., 2025 [8]. © 2025 Michael Meece, Shubham Rathore, Diego Zagazeta, and Elke K. Buschbeck. Originally published in Journal of Experimental Biology 228(6): jeb250043 (https://doi.org/10.1242/jeb.250043). Licensed under the Creative Commons Attribution 4.0 International License (CC BY 4.0). Changes were made to the original figure. B) Spectra of LED lighting used in the experiments. Damaging light consisted of two phases to maintain circadian entrainment: a 12 h day cycle, and a 12 h night cycle NIR light treatments involved 4 levels of intensity that were applied during two 30-minute time periods (morning and evening).

White mutant *Drosophila melanogaster* (w1118, Bloomington stock #3605) were fed ad libitum with Nutri-Fly Bloomington Formulation (Genesee Scientific, Morrisville, NC, USA) fly media with added molasses (35 mL per 1000 mL media). Freshly eclosed flies were sorted into two groups for 3 days of treatment. The first group was reared under typical indoor lighting from a white LED bulb (Ace A19 E26 6W LED bulb, Ace Hardware, Oak Brook, IL, USA) at ambient temperature and humidity and is hereafter referred to as the 3-day control (D3C) group. The second group, housed in an incubator at 22°C and ambient humidity, was exposed to constant, damaging bright light, also from white LEDs, for 72 hours and is hereafter referred to as the 3-day test (D3T) group. Following this initial 72-hour period, flies were either used to assess levels of damage or were placed into one of several recovery treatment groups. D3C flies were placed in identical conditions for an additional 10 days to serve as an age control for other 13-day-old treatment groups, hereafter termed 13-day control (D13C). D3T flies were either placed in conditions identical to those of D13C flies to serve as a gauge for baseline recovery (BLR) or they were placed in one of eight NIR-treated groups. NIR treatments consisted of 850 nm (ELD-850–525, Roithner LaserTechnik) and 950 nm (SIR-56ST3FF, Rohm Semiconductor, Kyoto, Japan) LEDs and had a target dose of 1, 4, 11, or 20 J/cm^2^. See Table 1 for LED intensity information and Fig 1 for a schematic of the treatment paradigm. All measurements of light intensity are given in photon/cm^2^/s, mW/cm^2^, and J/cm^2^ (for integrated measures of treatment dose).

**Table 1:** Summary information of LED intensity, treatment duration and overall dosage.

Treatment Group	Target Dose	Duration	Irradiance		3-day total fluence	30-minute fluence	10-day total fluence
	J/cm^2^		Photon/cm^2^/s	mW/cm^2^	J/cm^2^	J/cm^2^	J/cm^2^
3-day-old undamaged (D3C)		Day (12hr)	1.7x10^14^	0.058	7.5168	—	—
3-day-old damaged (D3T)		Day (12hr)	1.16x10^16^	4	518.4	—	—
—		Night (12hr)	1.18x10^15^	0.45	58.32	—	—
D3T cumulative		—	—	—	576.72	—	—
13-day-old control (D13C)		Ambient Lighting (12hr)	1.7x10^14^	0.058	—	—	—
Baseline recovery (BLR)		Ambient Lighting (12hr)	1.7x10^14^	0.058	—	—	—
850 nm-treated		Ambient Lighting (12hr)	1.7x10^14^	0.061	—	—	—
	1	NIR exposure (30 min)	1.18x10^14^	0.0275	—	0.0495	0.99
850 nm-treated		Ambient Lighting (12hr)	1.7x10^14^	0.059	—	—	—
	4	NIR exposure (30 min)	5.0x10^14^	0.116	—	0.2088	4.176
850 nm-treated		Ambient Lighting (12hr)	1.5x10^14^	0.053	—	—	—
	11	NIR exposure (30 min)	1.35x10^15^	0.314	—	0.5652	11.304
850 nm-treated		Ambient Lighting (12hr)	1.6x10^14^	0.056	—	—	—
	20	NIR exposure (30 min)	2.4x10^15^	0.557	—	1.0026	20.052
950 nm-treated		Ambient Lighting (12hr)	1.7x10^14^	0.059	—	—	—
	1	NIR exposure (30 min)	1.34x10^14^	0.0276	—	0.04968	0.9936
950 nm-treated		Ambient Lighting (12hr)	1.7x10^14^	0.061	—	—	—
	4	NIR exposure (30 min)	5.6x10^14^	0.113	—	0.2034	4.068
950 nm-treated		Ambient Lighting (12hr)	1.6x10^14^	0.056	—	—	—
	11	NIR exposure (30 min)	1.52x10^15^	0.311	—	0.5598	11.196
950 nm-treated		Ambient Lighting (12hr)	1.7x10^14^	0.059	—	—	—
	20	NIR exposure (30 min)	2.68x10^15^	0.553	—	0.9954	19.908

Values are shown in terms of quantum irradiance, irradiance, and energy density.

### Electroretinography

*Drosophila* were anesthetized and mounted for electroretinogram (ERG) recording as previously described by [10] and [25]. A 490 nm LED (LED490−06, Roithner Lasertechnik, Vienna, Austria) was used to deliver ERG stimuli at 4.28 × 10^10^, 8.15 × 10^10^, 2.78 × 10^11^, 7.2 × 10^11^, 1.75 × 10^12^, 5.42 × 10^12^, 1.04 × 10^13^, 5.53 × 10^13^ and 1.07 × 10^14^ photons cm^2^/s in order to define ERG response as a function of stimulus intensity (*v*log*I* curve). For each stimulus, 3 pulses of light were given with a 1 second pulse width and 15 seconds between pulses with 1 minute between each stimulus. The mean value for each train of 3 pulses was taken for analysis. Analyses were performed for ERG stimuli at 7.2 × 10^11^ and 1.07 × 10^14^ photons cm^2^/s. We specifically assessed the maximum photoreceptor response amplitude, on-transient response amplitude, and the time required for the field potential to return to 80% of the pre-stimulus baseline value after the termination of the ERG stimulus (hereafter termed recovery time). To put the on-transient amplitude and recovery time in perspective of the overall response, these values were normalized to the value of the photoreceptor response.

### Measurement of ATP concentration

ATP concentration was determined as described previously [8]. Flies were anesthetized on ice. Each sample consisted of five *Drosophila* heads, decapitated in 2.5% TCA. Heads were then placed in 100 µL 2.5% TCA and flash frozen in liquid nitrogen for 3 minutes. Samples were manually homogenized via 100 downward strokes with a micropestle. The supernatant was separated from the homogenate via centrifugation at 13,000 g for 15 minutes at 4°C. 10 µL supernatant was collected and diluted with 390 µL 1 M Tris-acetate buffer. L/rL reagent was added and luminescence was measured according to the manufacturer’s instructions using a BioTek Synergy 4 plate reader (BioTek, Winooski, VT, USA). ATP content per fly head was then calculated.

### Histology

To assess structural damage, we fixed bisected heads in a solution of 4% paraformaldehyde, 3.5% glutaraldehyde and 1% tannic acid in Sorensen’s phosphate buffer (all from Electron Microscopy Sciences, Hatfield, PA, USA) for 14-16h at 4°C. After thorough washing in Sorensen’s buffer, the tissue was incubated in 2% OsO_4_ for 2 hours (first hour in cold, second hour at RT), washed in dH_2_O and then incubated in 2% uranyl acetate for 14-16h at room temperature. The tissue was then dehydrated in a series of increasing ethanol and embedded in Ultra-Low Viscosity Embedding Medium (Polysciences, Warrington, PA, USA). Finally, the tissue was sectioned at 1µm with an Ultracut E Microtome (Reichert- Jung) and visualized with Nomarski optics on an Olympus BX51 microscope with a 100x Uplan objective. To correct for darkness gradients that are artifacts of the Nomarski optics, inverted Gaussian blur images were blended onto the original images (using the Soft light blending mode) and contrast and brightness were adjusted (all in Adobe Photoshop 2023).

### Statistical analyses

All statistical analyses were performed in RStudio (Posit, Boston, MA, USA). For all electroretinogram analyses (photoreceptor response amplitude, on-transient amplitude, and recovery time), a Kruskal-Wallis rank sum test was performed to determine inter-group differences. Multiple comparisons were then conducted with Benjamini-Hochberg corrected Dunn’s tests. For ATP content analysis, a two-way ANOVA was performed followed by Tukey HSD post-hoc multiple comparisons.

## Results

### Constant bright light exposure successfully reduces visual function

**Fig 2:**
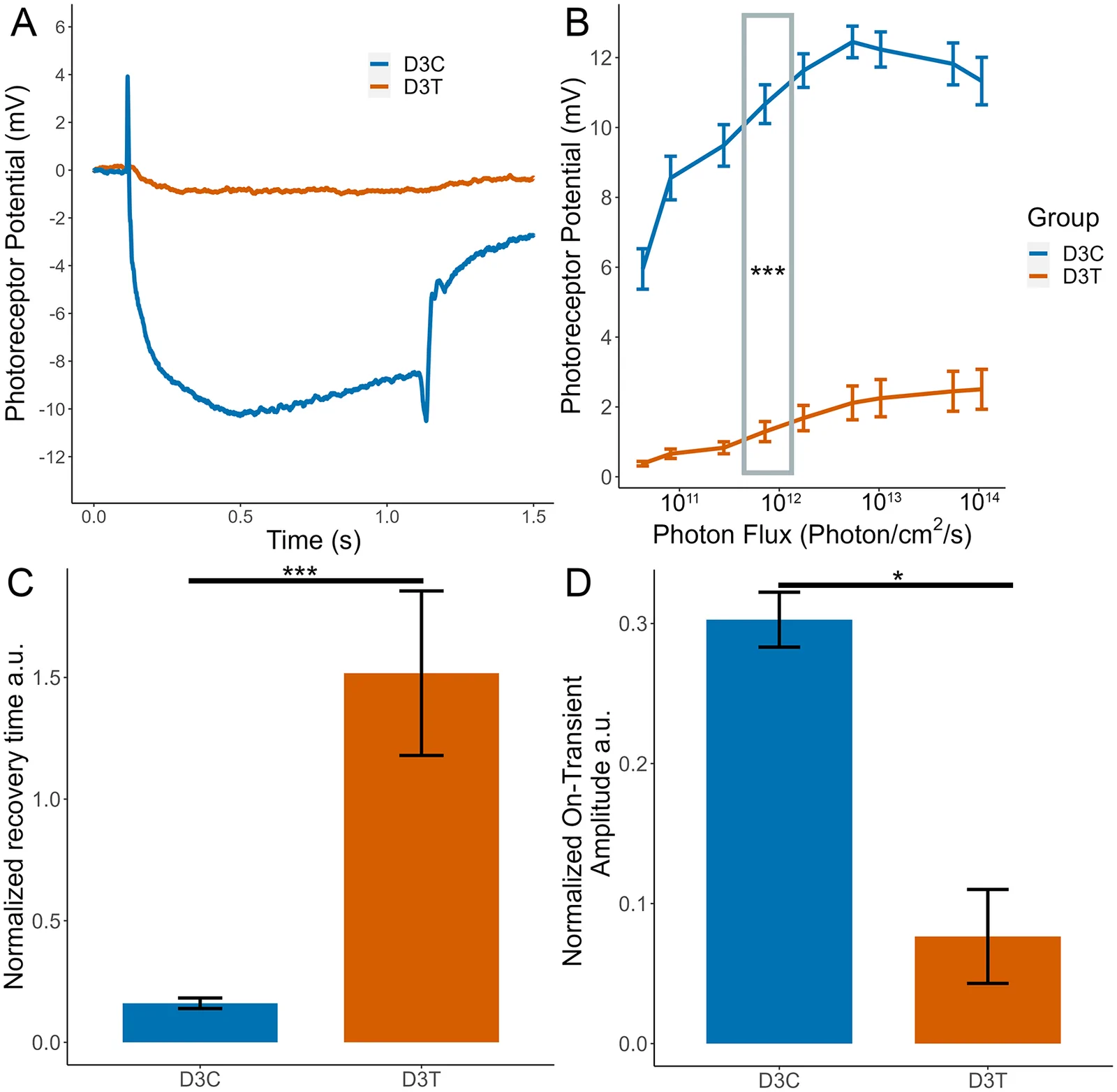
ERG responses in 3-day-old flies. A) Representative ERG traces of 3-day-old adult *D. melanogaster*. B) The *V*-log*I* curve of maximum photoreceptor potential for D3T (n = 27) and D3C (n = 28) flies illustrates how light-induced photoreceptor damage leads to a starkly reduced photoresponse (Dunn’s Test, Benjamini-Hochberg corrected, p = 2.14x10^-10^). C) When normalized to the magnitude of the photoresponse, the recovery time from light flashes (at 7.2x10^11^ photon/cm^2^/s) was significantly longer for photodamaged flies (Dunn’s Test, Benjamini-Hochberg corrected, p = 8.24x10^-4^). D) When normalized to the magnitude of the photoresponse, the on-transient response amplitude in response to at 7.2x10^11^ photon/cm^2^/s was significantly reduced in photodamaged flies (Dunn’s Test, Benjamini-Hochberg corrected, p = 0.014). In all cases error bars show standard error of the mean.

To evaluate recovery, it first has to be established that treatments cause photodamage. Indeed, following exposure to constant bright light for 72 hours, symptoms of retinal degeneration were evident, consistent with our previous work [8]. Electroretinograms (ERG) in D3T flies displayed a strongly reduced response, whereas the response of undamaged *Drosophila* were typical of the standard ERG waveform (Fig 2A). Photoreceptor response was heavily affected at all levels of intensity of light exposure (Fig 2B). For example, at a light intensity of 7.2x10^11^ photon/cm^2^/s (which is a good comparison value as it is within the linear range of the *V-*log*I* curve) D3C (n = 28) flies had a mean maximum photoreceptor response of 10.67 ± 0.55 mV, which was significantly higher than that of the D3T (n = 27) group which had a response of 1.29 ± 0.29 mV (Dunn’s Test, Benjamini-Hochberg corrected, p = 2.14x10^-10^). In addition, the recovery time, when normalized to the response magnitude, was notably longer in D3T flies (1.52 ± 0.34 a.u.) than in D3C flies (0.16 ± 0.02 a.u.)(Fig 2C; Dunn’s Test, Benjamini-Hochberg corrected, p = 8.24x10^-4^). It was also noted that only 24% of D3T flies, in contrast to 100% of D3C flies, displayed the on-transient response. When present, D3T flies (0.08 ± 0.03) had a lower normalized on-transient response amplitude (also normalized to the photoreceptor amplitude) than D3C flies (0.30 ± 0.02) (Fig 2D; Dunn’s Test, Benjamini-Hochberg corrected, p = 0.014).

### Visual function fails to recover without IR supplementation

**Fig 3:**
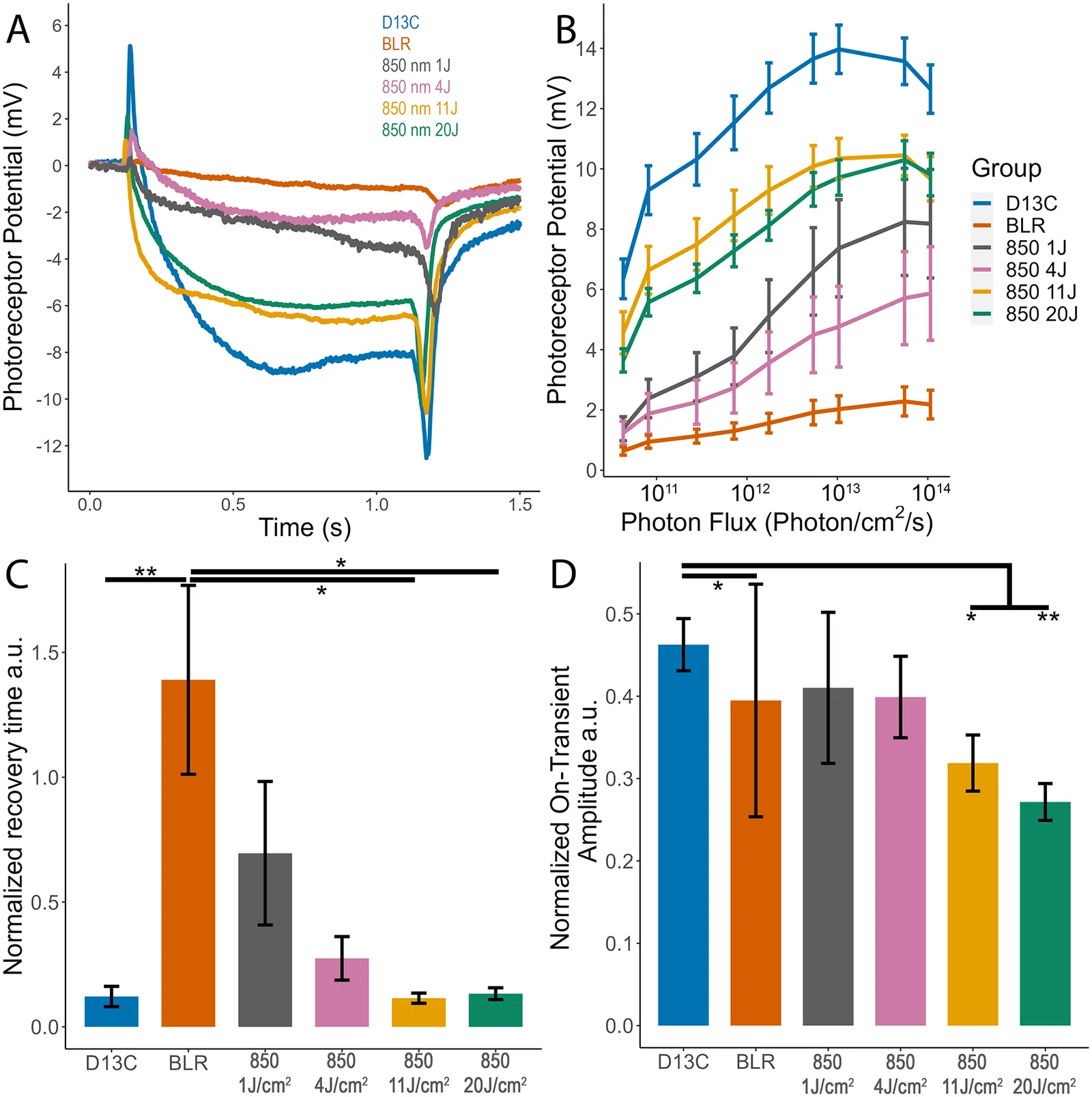
ERG responses in 850 nm treated flies. A) Representative ERG traces of 13-day-old adult *D. melanogaster* illustrating how higher levels of 850 nm IR light lead to improved recovery. Treatment with 850 nm light was given at total doses of 1 J/cm^2^ (n = 14), 4 J/cm^2^ (n = 13), 11 J/cm^2^ (n = 17), and 20 J/cm^2^ (n = 27). B) The *V*-log*I* curve of the maximum photoreceptor potential illustrates consistent differences throughout the dynamic range of the photoreceptors. Compared to D13C (n = 27) flies, the following groups had reduced photoreceptor responses: BLR (n = 33) (p = 7.35x10^-12^), 1 J/cm^2^ (p = 8.06x10^-4^), and 4 J/cm^2^ (p = 1.92x10^-5^). The following groups had photoreceptor response amplitudes greater than that of BLR flies: 1 (p = 0.033), 11 (p = 7.41x10^-7^), 20 J/cm^2^ (p = 2.51x10^-7^). C) Recovery times, normalized to the maximum photoresponse, following ERG stimulus at 7.2x10^11^ photon/cm^2^/s also are consistent with the highest light levels being most effective. BLR flies had a greater normalized recovery time than D13C, 11, and 20 J/cm^2^ (p = 0.0032, p = 0.014, and p = 0.012, respectively). D) The on-transient response amplitude, normalized to the maximum photoresponse after a 7.2x10^11^ photon/cm^2^/s light pulse however shows deficiencies in all treatment groups. D13C flies had greater normalized on-transient amplitudes than BLR, 11, and 20 J/cm^2^ flies (p = 0.023, p = 0.038, and p = 0.0012, respectively). In all cases error bars show standard error of the mean.

**Fig 4:**
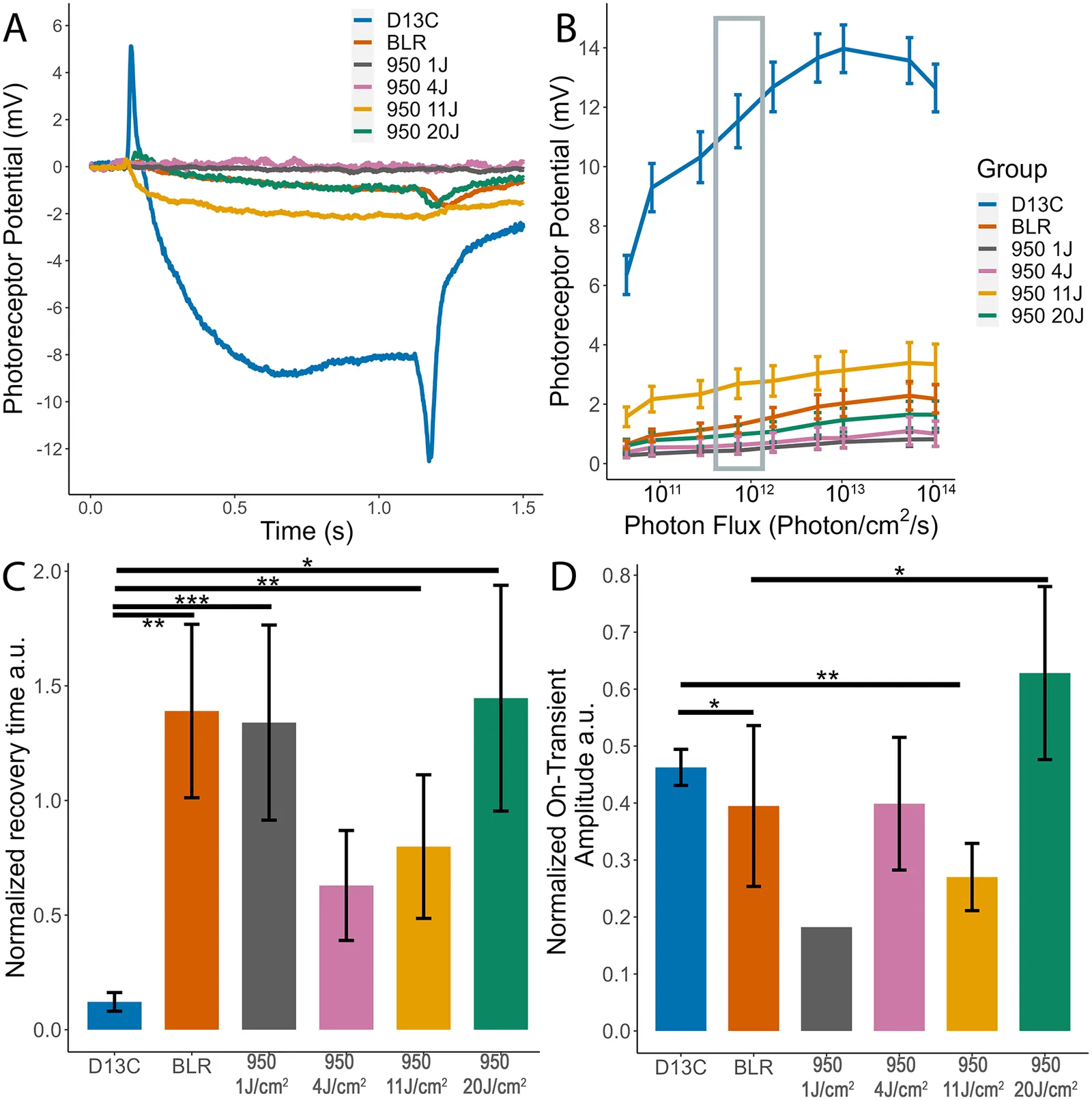
ERG responses in 950 nm treated flies. A) Representative ERG traces of 13-day-old adult *D. melanogaster* illustrates a strongly reduced photoreceptor response in all treatment groups. Treatment with 950 nm light was given at total doses of 1 J/cm^2^ (n = 16), 4 J/cm^2^ (n = 11), 11 J/cm^2^ (n = 18), and 20 J/cm^2^ (n = 20). B) The *V*-log*I* curve of the maximum photoreceptor potential illustrates that small responses are present at all stimulation intensities. All 950 nm-treated flies had smaller photoresponses than D13C flies (1 J/cm^2^: p = 4.16x10^-11^, 4 J/cm^2^: p = 1.02x10^-8^, 11 J/cm^2^: 1.98x10^-5^, 20 J/cm^2^: p = 8.42x10^-11^). C) The recovery time, normalized to the maximum photoresponse (following a light stimulus at 7.2x10^11^ photon/cm^2^/s) is generally relatively variable, with some dose dependence. D13C flies had lower recovery times than 1, 11, and 20 J/cm^2^ flies (p = 9.3x10^-4^, p = 0.0041, and p = 0.015, respectively). D) The on-transient response amplitude, normalized to maximum photoresponse, following a 7.2x10^11^ photon/cm^2^/s light pulse also shows great variability. 950 nm 11 J/cm^2^ treated flies had lower normalized on-transient amplitudes than D13C flies (p = 0.0015). 950 nm 20 J/cm^2^ treated flies had greater normalized on-transient amplitudes than BLR flies (p = 0.049). In all cases error bars show standard error of the mean.

To evaluate the recovery of bright-light induced damage, we assessed flies under different light conditions (Fig 1) 10 days post damage induction. The baseline recovery (BLR) group, which received 72 hours of damaging light exposure and was allowed to recover for 10 days in conditions identical to those given to D13C flies, showed no improvement in visual response when compared to D3T flies (Figs 3,4A). The BLR (n = 33) treatment group had a mean photoreceptor response of 1.30 ± 0.27 mV, which was significantly lower than that of the D13C (n = 27) treatment group (11.53 ± 0.89 mV)(Figs 3,4B; Dunn’s Test, Benjamini-Hochberg corrected, p = 07.35x10^-12^). Additionally, when normalized to the size of the photoreceptor response, BLR flies also had a longer recovery time (1.39 ± 0.38 a.u.) than D13C flies (0.12 ± 0.04 a.u.)(Figs 3 and 4C; Dunn’s Test, Benjamini-Hochberg corrected, p = 0.0032). 13-day-old flies also exhibited differences in the prevalence of the on-transient response: 92.6% of D13C flies and 36.3% of BLR flies presented the on-transient response. When present, D13C flies had a mean normalized on-transient amplitude of 0.46 ± 0.03 a.u. and BLR flies only had a mean normalized on-transient amplitude of 0.39 ± 0.14 a.u.(Figs 3 and 4D; Dunn’s Test, Benjamini-Hochberg corrected, p = 0.023).

### Recovery of visual function with 850 nm treatment is dose dependent

Consistent with our previous findings [8], treatment with 20 J/cm^2^ 850 nm light was observed to ameliorate the deterioration of visual function that was noted in BLR flies, raising the question as to what dosage might be most effective. Treatment with 850 nm light at total doses of 1 J/cm^2^ (n = 14), 4 J/cm^2^ (n = 13), 11 J/cm^2^ (n = 17), and 20 J/cm^2^ (n = 27) resulted in a photoreceptor response of 3.78 ± 0.94 mV, 2.73 ± 0.84 mV, 8.45 ± 0.84 mV, and 7.28 ± 0.53 mV, respectively, in response to 7.2x10^11^ photon/cm^2^/s light flashes. We found that higher doses, such as 11 J/cm^2^ and 20 J/cm^2^ were most effective. Notably, all flies that were treated with 850 nm light did not quite recover to the level of undamaged flies; 1 and 4 J/cm^2^ treated flies had photoreceptor responses lower than that of 13-day-old control flies (Dunn’s test, Benjamini-Hochberg corrected, p = 8.06x10^-4^ and p = 1.92x10^-5^, respectively). However, treatment with 1, 11, and 20 J/cm^2^ resulted in photoreceptor responses that were greater than those of BLR flies (Dunn’s test, Benjamini-Hochberg corrected, p = 0.033, p = 7.41x10^-7^, and p = 2.51x10^-7^, respectively), suggesting that all applied doses had some beneficial effects. Normalized recovery time also showed dose-dependence (Fig 3C). 11 J/cm^2^ and 20 J/cm^2^ treatment groups had relatively shorter recovery times than BLR flies with 0.11 ± 0.02 a.u. for 11 J/cm^2^ and 0.13 ± 0.02 a.u. for 20 J/cm^2^ (Dunn’s test, Benjamini-Hochberg corrected, p = 0.014 and p = 0.012, respectively). 1 J/cm^2^ treated flies had a mean recovery time of 0.70 ± 0.29 a.u., and the mean recovery time of 4 J/cm^2^ treated flies was 0.27 ± 0.09 a.u. The percentage of flies in each treatment group that had the on-transient response was 80% for 1 J/cm^2^, 61.5% for 4 J/cm^2^, 100% for 11 J/cm^2^, and 96.3% for 20 J/cm^2^ treated flies. The mean normalized on-transient amplitude for each 850 nm treated group was 0.41 ± 0.09 a.u. for 1 J/cm^2^, 0.40 ± 0.05 a.u. for 4 J/cm^2^, 0.32 ± 0.03 a.u. for 11 J/cm^2^, and 0.27 ± 0.06 a.u. for 20 J/cm^2^ treated flies (Fig 3D). Both 11 J/cm^2^ and 20 J/cm^2^ treated groups had lower normalized on-transient amplitudes than D13C flies (Dunn’s test, Benjamini-Hochberg corrected, p = 0.038 and p = 0.0012, respectively). None of the 850 nm treated groups had normalized on-transient responses that were significantly different from the BLR-treated group.

### 950 nm light is less effective than 850 nm light in recovery of visual function

Across all doses, 950 nm treatment was notably less effective than 850 nm light. The maximum photoreceptor responses of each 950 nm dose were 0.44 ± 0.12 mV for 1 J/cm^2^ (n = 16), 0.63 ± 0.29 mV for 4 J/cm^2^ (n = 11), 2.67 ± 0.50 mV for 11 J/cm^2^ (n = 18), and 0.98 ± 0.31 mV for 20 J/cm^2^ (n = 20). None of the doses showed improvement of the photoreceptor response compared with the BLR group, and all treatments resulted in photoreceptor responses that were significantly lower than that of D13C flies (Fig 4B; Dunn’s test, Benjamini-Hochberg corrected, 1 J/cm^2^: p = 4.16x10^-11^, 4 J/cm^2^: p = 1.02x10^-8^, 11 J/cm^2^: 1.98x10^-5^, 20 J/cm^2^: p = 8.42x10^-11^). Additionally, recovery time also did not improve following treatment with 950 nm light. 1 J/cm^2^ (1.34 ± 0.43 a.u.), 11 J/cm^2^ (0.80 ± 0.31 a.u.), and 20 J/cm^2^ (1.44 ± 0.49 a.u.) doses all had longer normalized recovery times compared to the D13C groups (Fig 4C; Dunn’s test, Benjamini-Hochberg corrected, p = 9.3x10^-4^, p = 0.0041, and p = 0.015, respectively). None of the treatments were statistically distinct from that of the BLR group; 4 J/cm^2^ treatment resulted in a normalized recovery time of 0.63 ± 0.24 a.u. The on-transient response was only observed in 6.25% (one individual) of 1 J/cm^2^, 4 36.4% of J/cm^2^, 11 77.8% of J/cm^2^, and 25% of 20 J/cm^2^ treated flies. When present, the mean normalized amplitude of the on-transient response for each treatment group was 0.18 a.u. for 1 J/cm^2^, 0.40 ± 0.12 a.u. for 4 J/cm^2^, 0.27 ± 0.06 a.u. for 11 J/cm^2^, and 0.63 ± 0.15 a.u. for 20 J/cm^2^ (Fig 4D). Flies in the 11 J/cm^2^ treatment group had a normalized on-transient amplitude that was smaller than that of the D13C group (Dunn’s Test, Benjamini-Hochberg corrected, p = 0.0015) and those treated with 20 J/cm^2^ had an on-transient amplitude greater than that of the BLR group (Dunn’s test, Benjamini-Hochberg corrected, p = 0.049).

### Histological comparison reveals substantial damage in all treatments

**Fig 5:**
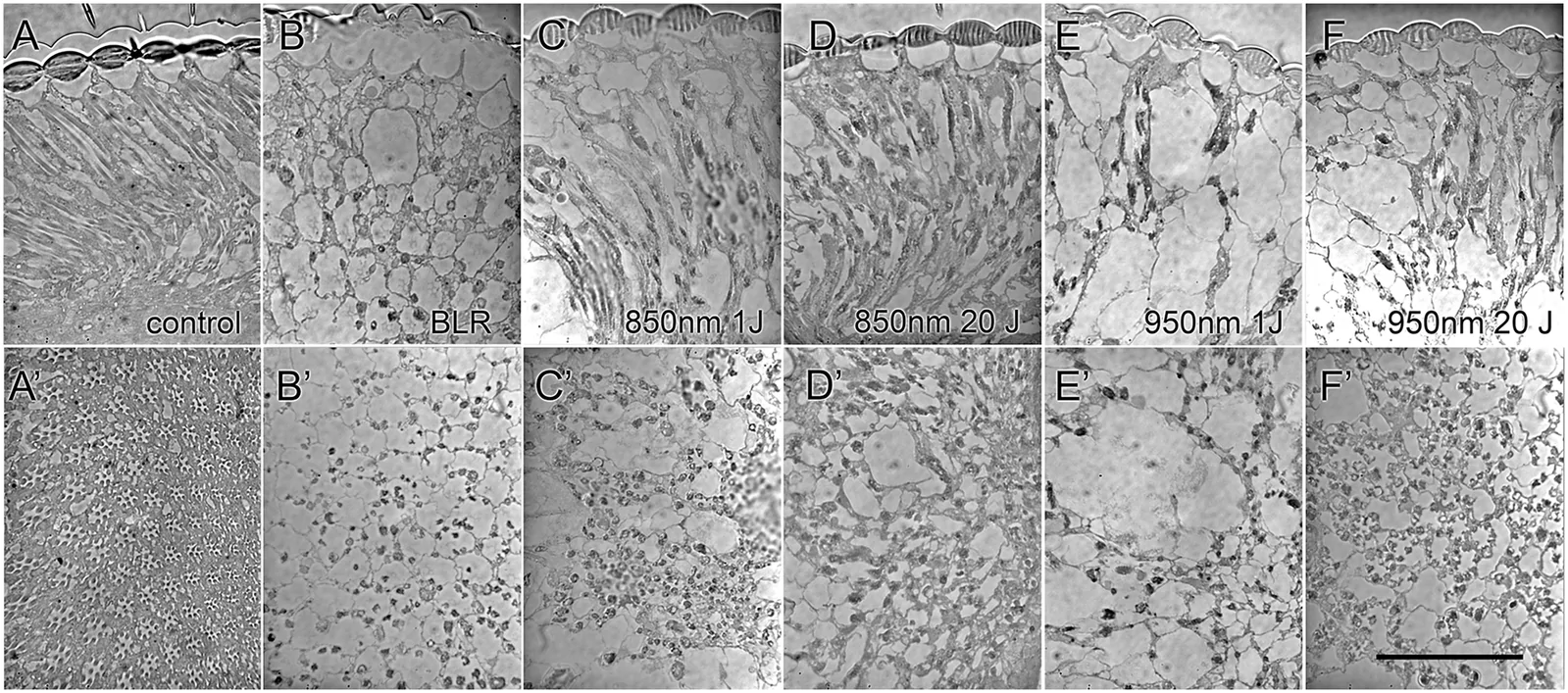
Histological assessment of eye tissue. Length (A) and cross (A’) sections of eyes illustrate the relatively intact eye tissue of control animals. In stark contrast length (B) and cross (B’) sections of light damaged flies that recovered under control lighting shows substantially damaged tissue. Likewise substantially damaged are all light recovery groups with apparent slightly better recovery of the 860nm (C, C’ D & D’) light exposed flies compared to the 950nm (D, D; E & E’) exposed flies (C-E represents length and C’-E’ cross sections). All images are at the same scale with the scale bar representing 500µm.

To evaluate how light treatments affected the structure of the visual system, we performed histological sections of eye tissue (Fig 5)**.** Apart from the control animals, all groups revealed substantial damage in the structural components of the ommatidia. The damage is clearly apparent in both, length and cross sections of the ommatidial array and is so substantial that in many cases rhabdomes are not visible at the light microscopical level.

### NIR dose affects ATP concentration

**Fig 6:**
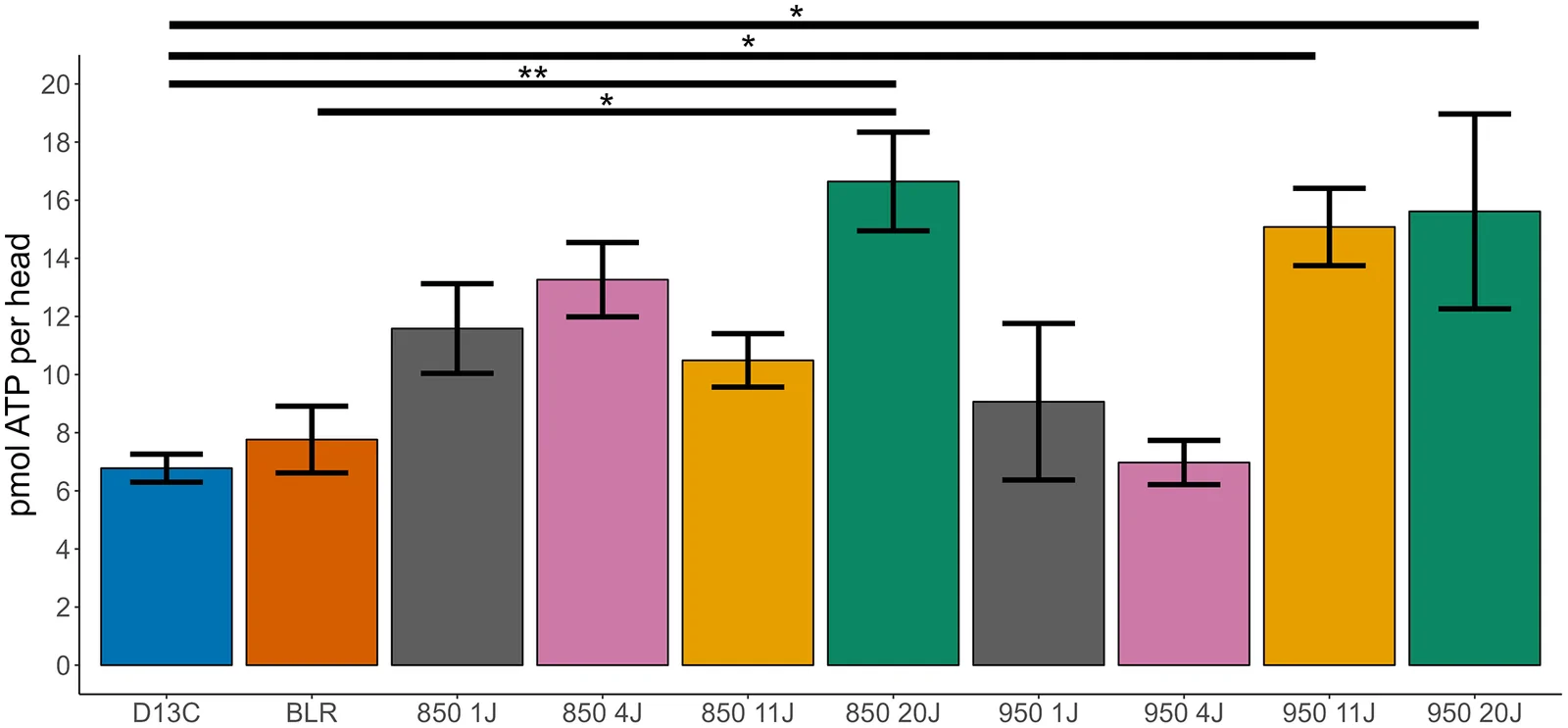
ATP content in *Drosophila* heads. 13 day old control flies (n = 7) and flies that were allowed to recover in background lighting (BLR; n = 6) show comparable ATP levels. Treatment with 850 nm light leads to somewhat elevated ATP levels at all exposure levels, with a significant increase at the highest light intensity. Sample sizes were as follows: 1 (n = 8), 4 (n = 7), 11 (n = 7), and 20 J/cm^2^ (n = 7). Treatment with 950 nm light leads to little differences in ATP levels at lower light levels, but to a significant increase at the two highest treatment dosages. Sample sizes were as follows: 1 (n = 5), 4 (n = 8), 11 (n = 8), and 20 J/cm^2^ (n = 6). 850 nm 20 J/cm^2^, 950 nm 11 J/cm^2^, and 950 nm 20 J/cm^2^ treated flies had greater ATP concentrations than D13C flies (p = 0.0016, p = 0.01, and p = 0.011 respectively). 850 nm 20 J/cm^2^ treated flies had greater ATP concentrations than BLR flies (p = 0.011). In all cases, error bars represent standard error of the mean.

Despite stark physiological differences between the two wavelengths, ATP levels improved with both wavelengths. Based on our statistical analysis, treatment with NIR light was found to affect the measured ATP content of dissected *Drosophila* heads (Fig 6; ANOVA, F_9_ = 5.45, p = 1.97x10^-5^). D13C (n = 7) and BLR (n = 6) flies had observed ATP contents of 6.78 ± 0.48 pmol/head and 7.76 ± 1.15 pmol/head, respectively. All flies treated with 850 nm light showed elevated values and *Drosophila* treated with the 20 J/cm^2^ dose had significantly greater ATP content than either D13C (Tukey HSD, p = 0.0016) or BLR flies (Tukey HSD, p = 0.011). Specifically the mean ATP contents were 11.58 ± 1.54 pmol/head for 1 J/cm^2^ (n = 8), 13.26 ± 1.28 pmol/head for 4 J/cm^2^ (n = 7), 10.49 ± 0.92 pmol/head for 11 J/cm^2^ (n = 7), and 16.64 ± 1.70 pmol/head for 20 J/cm^2^ (n = 7). 950 nm treatments also resulted in elevated ATP values, but only at relatively high light levels. Specifically the ATP content was 9.07 ± 2.70 pmol/head for 1 J/cm^2^ (n = 5), 6.97 ± 0.76 pmol/head for 4 J/cm^2^ (n = 8), 15.08 ± 1.33 pmol/head for 11 J/cm^2^ (n = 8), and 15.6 ± 3.35 pmol/head for 20 J/cm^2^ (n = 6) doses. For both 11 J/cm^2^ and 20 J/cm^2^ treated flies these ATP values are significantly higher than those of the D13C flies (Tukey HSD, p = 0.01 and p = 0.011, respectively).

## Discussion

The aim of this study was to resolve specific efficacies of different doses of the previously applied wavelengths of near-infrared (NIR) and infrared (IR) light in the treatment of light-induced retinal damage in *Drosophila melanogaster* [8]. In this study, we observed a wavelength-specific effect of PBM treatment on retinal damage (at equal photon flux), with 850 nm treatment leading to a far better recovery of the photoreceptor response than 950 nm. However, because photons of different wavelengths carry different energy, keeping photon flux equal necessarily meant that the shorter wavelength was applied with greater energy, leaving the possibility that observed effects could have been due to differences in energy density. To follow up on this question and expand our understanding of how to dose NIR and IR light, we tested the effects of these two wavelengths at different doses (fluences). Specifically we focused on intensity levels that have already been applied in photobiomodulation studies (for review see [2]), as well as at complementary doses to those used in [8]. For example, we included 11 J/cm^2^ for 850 nm to be able to compare it to the previously tested 11 J/cm^2^ 950 nm light, and 20 J/cm^2^ for 950 to be comparable to the previously tested 20 J/cm^2^ 850 nm light treatment. Our findings reveal that all 850 nm light intensities lead to improved retinal function when compared to 950 nm, with the higher light intensities showing stronger effects.

As in the previous study, toxic levels of bright light led to greatly impaired photoreceptor responses, leading to persistent retinal damage that failed to recover on its own. To explore such damage further, we incorporated an analysis of the recovery period of stimulated photoreceptors, with damaged photoreceptors showing a longer recovery time relative to their much smaller photoreceptor responses (Fig 4). This could suggest a reduced efficiency of the processes involved in repolarizing photoreceptors [26–29]. The amplitude of the on-transient response was also lower in light-damaged flies, indicating a detrimental effect on synaptic health at the level of the photoreceptor or an otherwise reduced capacity of the retina to transmit signal to the lamina [30, 31].

### Dose-specific effects

**Fig 7:**
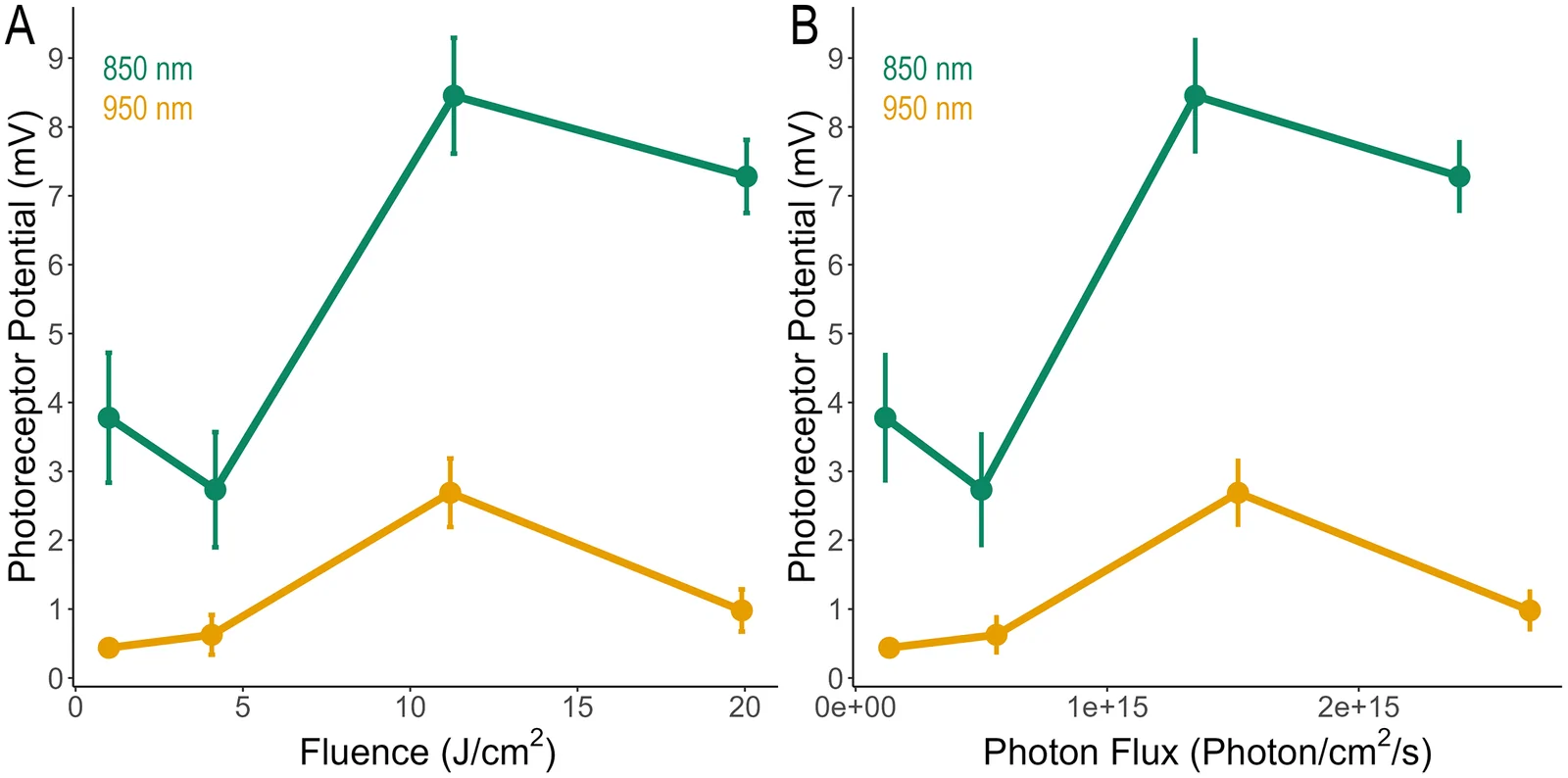
Direct comparison between Fluence and Photon Flux. The maximum photoreceptor response to a light flash of 7.2x10^11^ photon/cm^2^/s for both 850 nm- (green) and 950 nm-treated (yellow) is illustrated in two different ways to allow for direct comparison between fluence (A) and quantum irradiance (B). As photons of shorter wavelengths carry more energy, measurements are shifted relative to each other.

Treatment with 850 nm light was found to be especially effective in promoting the recovery of damaged retinae in *Drosophila melanogaster* at relatively high doses, namely 11 and 20 J/cm^2^. However, even the photoresponses of 1 and 4 J/cm^2^ treated flies showed some benefits, especially when measured at higher intensities of light stimuli (Fig 3B). This finding is in line with others (see [2] for a review of relevant literature) suggesting that ineffective treatment with PBM usually stems from underdosing rather than overdosing. In [8], the intensity of the 850 nm treatment used is comparable to the 20 J/cm^2^ dose described in this study. Although we generally were able to reproduce previous findings, small differences may relate to a minor change in the application of the damaging light or batch effects arising due to biological variability in flies. Specifically, the 72-hour damaging treatment for D3C, BLR, and PBM-treated groups ended up being slightly brighter in this study, possibly leading to slightly higher levels of damage, which also could potentially explain the slightly reduced response after recovery in the 20 J/cm^2^ light treatment. Even with this caveat, the most effective treatment for our paradigm was 11 J/cm^2^ (Fig 7). The notion that our damaging light caused particularly dramatic damage also is supported by our histological investigation that showed substantial damage in all groups (Fig 5). Consistent with the physiology, the 850nm treated groups appeared to recover slightly better than the 950nm treated groups. However to determine if that indeed is the case, a more detailed quantitative analysis is necessary, and a follow-up study at the ultrastructural level is forthcoming [32]. As a whole, treatment with 950 nm light was less effective and notably, irrespective of dosage, none of the 950 nm treatments significantly improved physiological recovery compared to the BLR group. We also observed that the photoreceptor response to increasing intensities of light for 950 nm treated flies was less dynamic than that of undamaged or 850 nm-treated flies. While not resulting in significant improvement, flies treated with a fluence of 11 J/cm^2^ had the best recovery of all 950 nm-treated flies (Fig 7). This suggests that in our paradigm this may be the optimal dose as it was most effective at both 850 and 950 nm.

We defined photoreceptor recovery time (PRT) as the time taken for photoreceptor potential to return to baseline after each light pulse. For 850 nm, we found that treatments with higher doses resulted in relatively shorter PRT, with times to return to baseline that are comparable to those of undamaged flies. However, in flies treated with 950 nm light, only those treated with 4 and 11 J/cm^2^ had slightly reduced normalized PRT, albeit not significantly. It is possible that in flies with reduced PRT, photoreceptors are generally healthier than in those with increased PRT. While much focus is being put on photoreceptors responding to light, it is equally important for them to be “turned off” again. These steps include the phosphorylation of metarhodopsin by the G protein–coupled receptor kinase 1 as well as binding of Arrestin, which in itself is modulated by available Ca^2+^ [27, 33, 34]. It is possible that an increase in the time required to repolarize the cell is indicative of a reduced efficacy of these or other cell processes that are involved in cell repolarization [26, 28], a topic that warrants further investigation. Another way to evaluate the integrity of the retinal circuitry is to examine the presence and strengths of the on-transient, which correspond to the synaptic activation of first-order interneurons [31]. Because a stronger photoresponse also tends to more strongly activate the first-order synapse, here we look at the on-transient relative to the photoreceptor response (see S1 Fig for absolute values). A higher signal is interpreted as indicative of a relatively upregulated synaptic connection, which already is known to occur when the signal is weak [30]. Interestingly, flies treated with 850 nm light at 11 or 20 J/cm^2^ (which had the strongest recovery of the photoreceptor signal itself) had relatively reduced on-transient responses when compared to undamaged flies. This is despite the fact that in absolute terms their on-transients were larger than those of the flies that had recovered under 1 J/cm^2^ and 4 J/cm^2^ light (See S1 Fig). This may suggest that other parts of the retinal circuitry of these flies may not have fully recovered, despite the strong photoreceptor response. For 950 nm light, the 11 J/cm^2^ dose, but not the 20 J/cm^2^ dose, led to reduced relative on-transient amplitude. In contrast, flies treated with 20 J/cm^2^ of 950 nm light had relatively larger on-transient amplitudes than the BLR flies. This implies that the downstream signal is upregulated relative to the very weak response of the photoreceptors. The high intensity 950 nm treatment instead could have some beneficial effects, but rather than improving the photoreceptor health directly this would be mediated by upregulating the downstream signal. Taken together, there is some variability in the size and occurrences of on-transients, with relatively larger on-transients being associated with smaller photoreceptor responses at least for some flies. This is consistent with plasticity in that circuit [35]. Since different mechanisms govern the photoreceptor response and the on-transient, our data offer insight into how various light treatments affect these processes.

Our assessment of ATP levels suggests that, as in other PBM studies [36], ATP may play a major role in the recovery of the tissue. Specifically we found that all doses of 850 nm light treatment resulted in increased ATP concentration, with the 20 J/cm^2^-dosed group showing a statistically significant difference, with more than doubled ATP level. Interestingly, flies treated with 950 nm light had increased ATP levels at doses of 11 and 20 J/cm^2^ yet neither of these groups recovered in terms of their photoreceptor function. This suggests that while increased ATP availability likely is indicative of improved metabolic functions, it in itself is not enough to promote the recovery of the damaged retina.

### Broader significance

Our study clearly shows that 850 nm is more effective than 950 nm light in promoting photoreceptors to recover from light-induced damage, and that this is the case irrespective of a wide variety of dosages. For lower intensities, 850 nm light also has been more effective in promoting increased levels of ATP. Notably, here we did not find a direct correlation between increased ATP levels and the recovery of the photoreceptor response, suggesting that multiple processes may be involved. Some clues may come from a previous investigation into the structural integrity of treated photoreceptors. Specifically, based on an evaluation of the autofluorescence [37, 38] we previously found that in contrast to the light-damaged flies, those flies treated with 950 nm light at ≈11.5 J/cm^2^ fluence had structurally relatively intact photoreceptors [8] despite major deficits in the physiology. In the current study we find relatively intact physiology, despite major structural deficits. Therefore, it is possible that elevated ATP levels help recovery in multiple ways, with structural and physiological recovery being relatively uncoupled. Such an interpretation is feasible, as a previous study in *Drosophila melanogaster* has demonstrated that gene knockdown can result in intact retinal morphology despite severely impaired ERG responses, while conversely, severely disrupted eye morphology can still be associated with normal ERG responses.[10]. Increased ATP production is one way in which photobiomodulation is known to work, but it likely also affects the efficiency of the process and related production of toxic ROS [4]. For example, a study in which mitochondrial function was evaluated after relatively short exposure (15 min) to harmful blue light, showed significant reduction in some of the mitochondrial complexes while still maintaining near normal ATP production [39]. A more prolonged light stimulation however did lead to reduced ATP levels. A more detailed evaluation of mitochondrial function will be necessary to clarify how 850 nm versus 950 nm light may differentially influence specific aspects of the very complex metabolic pathway that takes place in the mitochondria. This includes evaluation of ROS, increased levels of which are known to affect the cell as a whole and damage the structural integrity of many cellular components, including proteins and the lipid bilayer [40]. Another possible mechanism involves heat. It has been previously shown that longer wavelengths of NIR light (≈980 nm) are absorbed by water, which could then locally alter the temperature and affect associated processes. For example, it has been demonstrated that such an effect can influence the amount of available calcium (Ca^2+^) in the cytoplasm [41, 42]. While this mechanism is thought to positively affect metabolic activity, the temperature sensitive TRP and TRPL channels in the *Drosophila* photoreceptor are known to be strongly inhibited by the presence of Ca^2+^ [27]. While purely hypothetical at this point, this could potentially explain the reduced photoreceptor response observed in 950 nm-treated flies, despite elevated levels of ATP and increased metarhodopsin present in 950 nm-treated flies in [8]. However, it is worth noting that it has previously been found that optimal dosage can be highly wavelength specific. In one study the effective dose for a longer wavelength (980 nm) was much (10–100 fold) lower than for a shorter wavelength (810 nm) [41], so it is possible that there are benefits of 950 nm light that are outside the parameter space that we investigated.

Important next steps include a deeper analysis of certain mitochondrial processes. For example, specific effects on mitochondrial activity could be assessed, as has previously been done in other PBM studies [4, 43]. A study in aging *D. melanogaster* demonstrating beneficial effects of red light treatment [44] also raises the intriguing possibility that 850nm illumination could confer similar or even enhanced benefits in this context. To evaluate structural integrity, a detailed ultrastructural analysis of photoreceptor rhabdomeres and photoreceptor-associated mitochondria under different light treatments would be beneficial. Such an analysis may be particularly interesting to elucidate why treatments with 950 nm light are relatively unsuccessful despite elevated ATP levels. Taken together, this and related studies highlight the potential that the field of photobiomodulation has to offer. While it has become more apparent in recent years that PBM can be effective, many more comparative studies focusing on different illumination parameters are needed to better understand how PBM effectively allows treating damaged tissues.

### Highlights

- This study uses *D. melanogaster* eyes to systematically compare optimal dosages of two wavelengths that were found to be biologically significant, in promoting the recovery of previously damaged photoreceptor cells.
- We quantify our light treatments with spectra, photon counts and the more conventional total energy (fluence). This sets an important trend towards making photobiomodulation studies more comparable.
- We identify distinct physiological and ATP-related effects that suggest that multiple mechanisms may be contributing to observed neuronal recovery.

## Supporting information

S1 Fig Absolute on-transient amplitude. The on-transient amplitudes of D3C and D3T flies (A), and 13-day-old D13C, BLR, 850 nm-treated, and 950 nm-treated flies (B).
(TIF)

S1 Dataset The original data set that was used for the analysis. (XLSX)
